# Incorporation of a truncated form of flagellin (TFlg) into porcine circovirus type 2 virus-like particles enhances immune responses in mice

**DOI:** 10.1186/s12917-020-2253-6

**Published:** 2020-02-07

**Authors:** Xiangyu Liu, Yangkun Liu, Yuanyuan Zhang, Fan Zhang, Enqi Du

**Affiliations:** College of Veterinary Medicine, Northwest A&F University, Yangling, Shaanxi People’s Republic of China

**Keywords:** Flagellin, Porcine circovirus type 2, Virus-like particles, *Escherichia coli*

## Abstract

**Background:**

Porcine circovirus type 2 (PCV2) is an economically important pathogen in the swine industry worldwide. Vaccination remains the principal tool to control PCV2-associated diseases (PCVADs). Current vaccines do not eliminate viral shedding in the environment. To enhance the efficacy of PCV2 vaccines, recombinant virus-like particles (VLPs) of PCV2 were generated by fusing a truncated form of flagellin FliC (TFlg: 85-111aa) with the PCV2 capsid protein (Cap).

**Results:**

The recombinant proteins were expressed in *Escherichia coli* and detected using Western blotting. The abilities of the recombinant proteins to assemble into VLPs were observed under transmission electron microscopy (TEM). The protective immune responses of recombinant VLPs were further evaluated by immunization of mice. The results showed that insertion of TFlg into C terminal of the Cap protein did not affect the formation of VLPs and boosted both humoral and cellular immune responses in mice. After a challenge with PCV2, in the Cap-TFlg vaccinated group, viremia was milder and viral loads were lower as compared with those in the Cap vaccinated group.

**Conclusion:**

These results suggest that recombinant VLPs of PCV2 containing a TFlg adjuvant can be used as a promising PCV2 vaccine candidate.

## Background

Porcine circovirus type 2 (PCV2) is considered a pivotal pathogen of PCV2-associated diseases (PCVADs), which have a serious impact on the swine industry worldwide [1]. PCV2 contains three major open reading frames (ORFs), which encode a replicase protein, viral structural capsid (Cap) protein, and viral pathogenesis-associated protein [2] Among these proteins, the Cap protein is the primary immunogenic protein and thus has been a target in studies aimed at developing new vaccines against PCVADs [3, 4].

Virus-like particles (VLPs) are highly effective vaccine candidates, as they mimic the structure of native viruses. VLP-based vaccines can stimulate strong B-cell mediated responses and cytotoxic T-lymphocyte responses. In comparison with monomeric antigens, lower doses of VLPs can induce a similar level of immune response [5, 6]. These advantages have made VLPs potential vaccine candidates for many viral diseases. Recently, the potential of multiple expression systems, including *Escherichia coli*, baculovirus, and yeast, in producing a PCV2 VLP-based vaccine has been explored [7–9]. Research reported that vaccines based on VLPs were effective in reducing mortality and morbidity and improving productive performances, although they did not provide complete protection against PCV2 infection [10]. Therefore, methods to enhance the efficacy of PCV2 VLP-based vaccines are desirable.

Flagellin, the primary protein component of bacterial flagella, has been widely used as a potent adjuvant to induce both systemic and mucosal immune responses [11]. The immunomodulatory effects of flagellin proteins are mediated mainly by activating toll-like receptor 5-positive immune cells, especially DCs [12, 13]. To date, studies have shown strong adjuvant effects of flagellins of *Salmonella typhimurium* (FliC and FljB) in many vaccine candidates against *Yersinia pestis* [14, 15], *Plasmodium falciparum* [16], *Clostridium tetani* [17], influenza [18], and West Nile viruses [19]. Moreover, a previous report showed that the 9 flagellin-related peptides (9Flg), which contains amino acids 85–111 of the mature flagellin FliC, can be used as an adjuvant to enhance antigen-specific immunity in vitro and in vivo [20]. This evidence strongly suggests that truncated form of flagellin (TFlg) may act as a broad adjuvant in vaccines. However, no evidence on adjuvant effects of TFlg in pig vaccines has been reported. Therefore, the present study examined whether TFlg enhanced immune immunity conferred by the PCV2 VLP-based vaccine.

In the present study, we report for the first time insertion of TFlg into C terminal of PCV2 Cap protein to generate recombinant VLPs *in E. coli.* The recombinant Cap-TFlg proteins self-assembled into VLPs. In addition, TFlg enhanced both humoral and cellular immune responses, provided protection against PCVAD, and promoted vaccine efficiency after vaccination.

## Results

### Production of cap and cap-TFlg proteins in *E. coli*

**Fig. 1:**
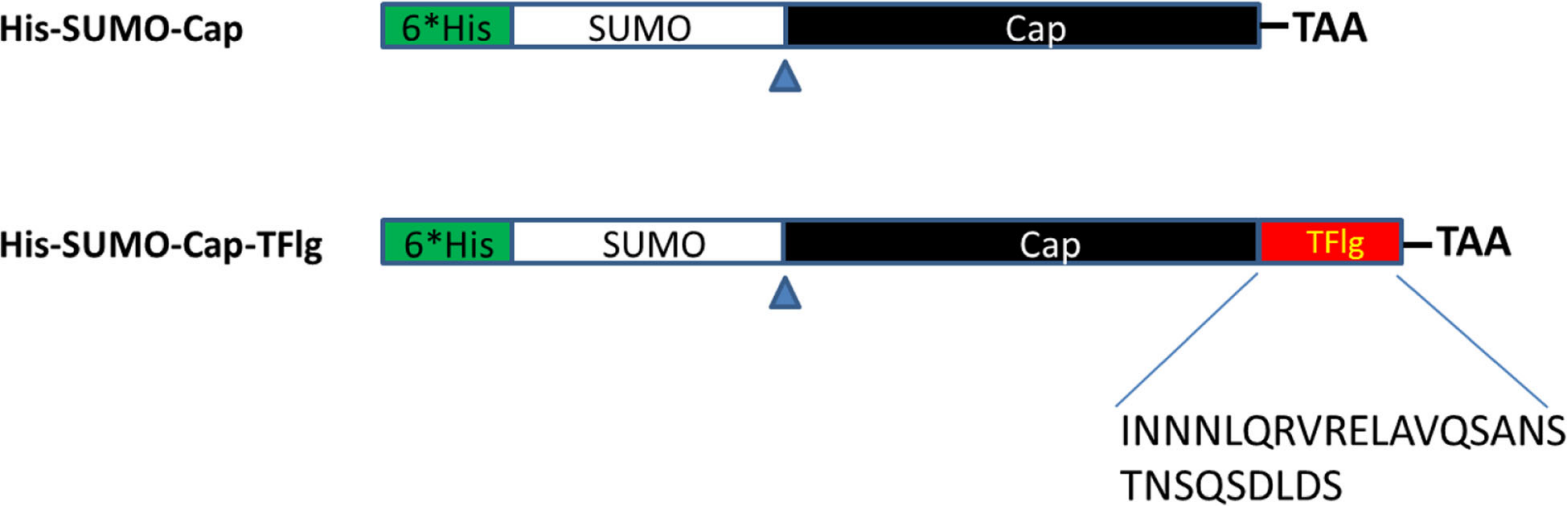
Schematic representation of the SUMO-Cap and SUMO-Cap-TFlg fusion genes in this study. A 6 × His tag has been added to the N-terminal of the SUMO protein, and the stop codon TAA has been added in front of the Xho I restriction enzyme site. Arrowheads indicate the SUMO protease cleavage site

The genes encoding SUMO-Cap and SUMO-Cap-TFlg were inserted into plasmid pET32a as described above (Fig. 1).

**Fig. 2:**
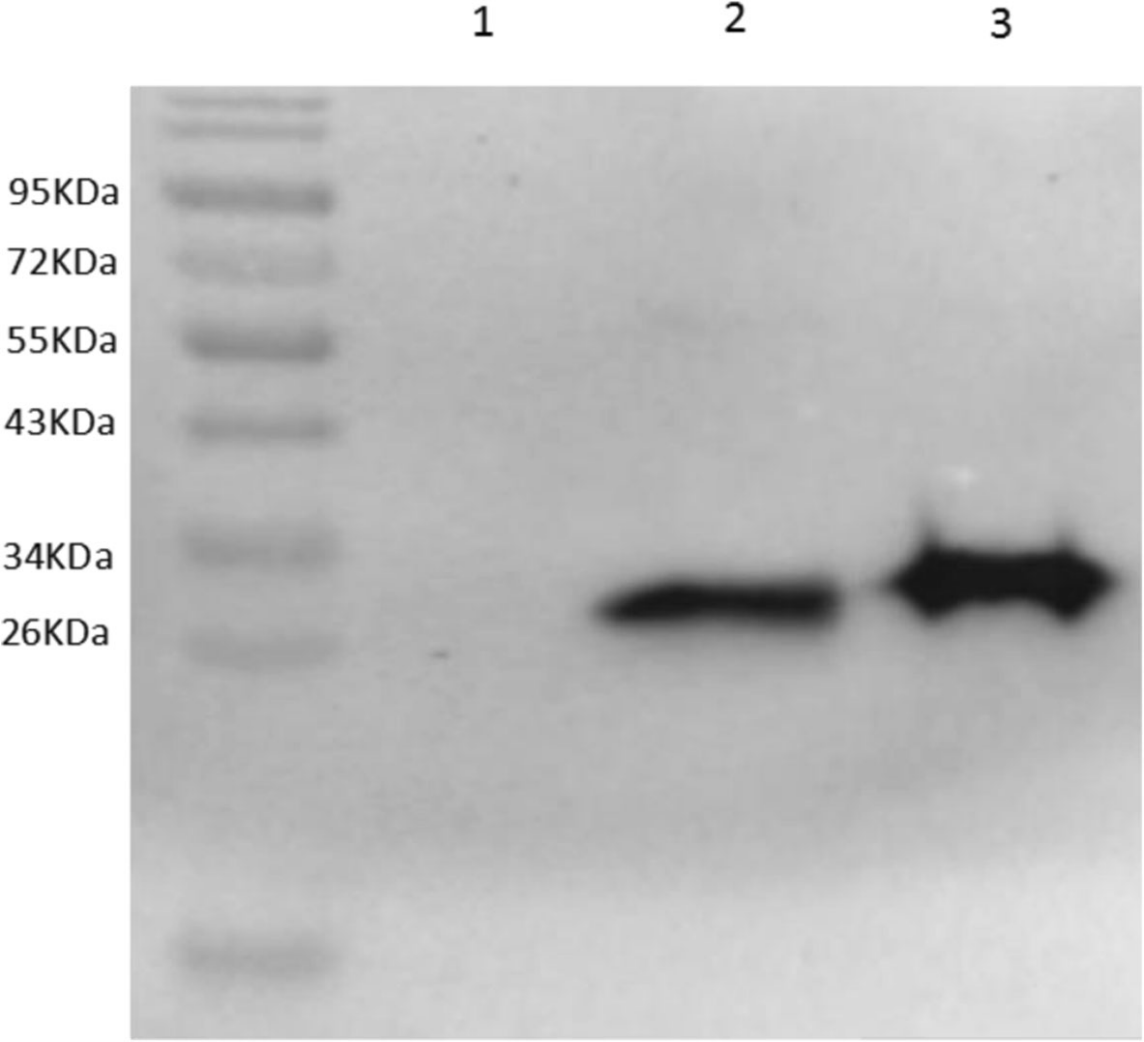
Detection of purified recombinant proteins by western blotting with rabbit anti-Cap antibody. Lane 1:negative control. Lane 2: Cap-TFlg.3:Cap

The recombinant plasmids were verified by DNA sequencing and then transformed into *E. coli* BL21 (DE3) for protein expression. In addition, a 6 × His tag was fused upstream of the SUMO tag to allow purification of the fusion protein using Ni-NTA affinity chromatography. A typical procedure for purification of the Cap and Cap-TFlg proteins is illustrated in Fig. 2a. Finally, the purified Cap protein (about 28 kDa) and Cap-TFlg protein (about 31 kDa) were confirmed by Western blotting. The reaction of Cap or Cap-TFlg protein with rabbit anti-Cap antibody was detected by Western blotting (Fig. 2b).

### Transmission electron microscopy (TEM) analysis

**Fig. 3:**
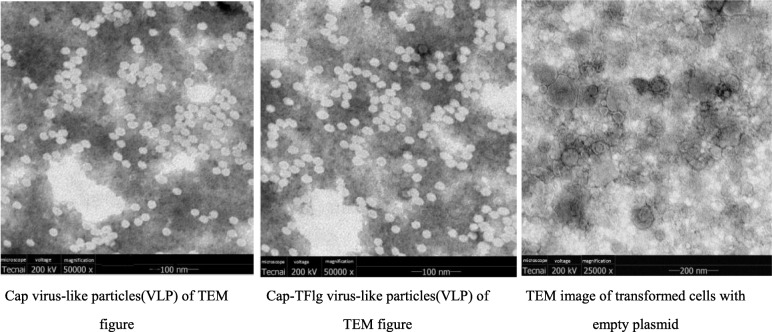
Virus-like particles (VLP) observation by transmission electron microscopy

To test whether the purified Cap and Cap-TFlg protein assembled into VLPs, the proteins were observed by TEM. The results showed that the purified Cap-TFlg proteins self-assembled into VLPs, with sizes and morphologies similar to those of PCV2 Cap VLPs, which had a diameter of 17–20 nm (Fig. 3).

### PCV2-specific humoral immune responses

**Fig. 4:**
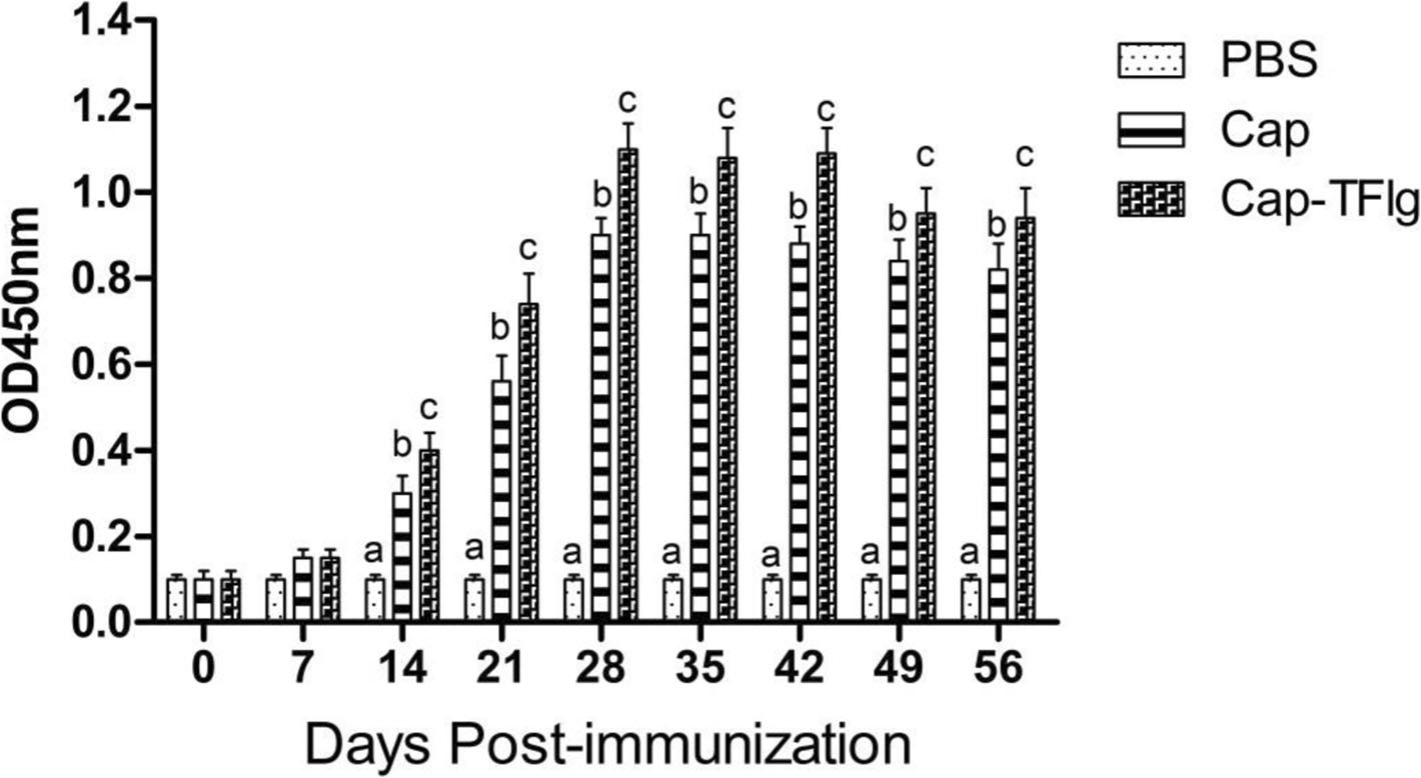
PCV2-specific serum antibodies detected by indirect ELISASerum samples of mice (*n* = 5 per group) were harvested at various time points post primary immunization to detect the PCV2-specific antibodies. Data are shown as mean ± SD of triplicate measurements. The data sets labeled with different superscript letters (a, b, and c) are statistically different from each other (*P* < 0.05)

As shown in Fig. 4, the PCV2-specific antibodies first appeared at 14 dpi in Cap and Cap-TFlg vaccinated groups, and the antibody titers then increased rapidly to a peak at 28 dpi. The PCV2-specific antibody titer in mice in the Cap-TFlg vaccinated group was significantly higher than that of the Cap vaccinated group after 14 dpi (*P* < 0.05). In contrast, the mice inoculated with PBS did not develop detectable antibody titers during the experiment.

**Fig. 5:**
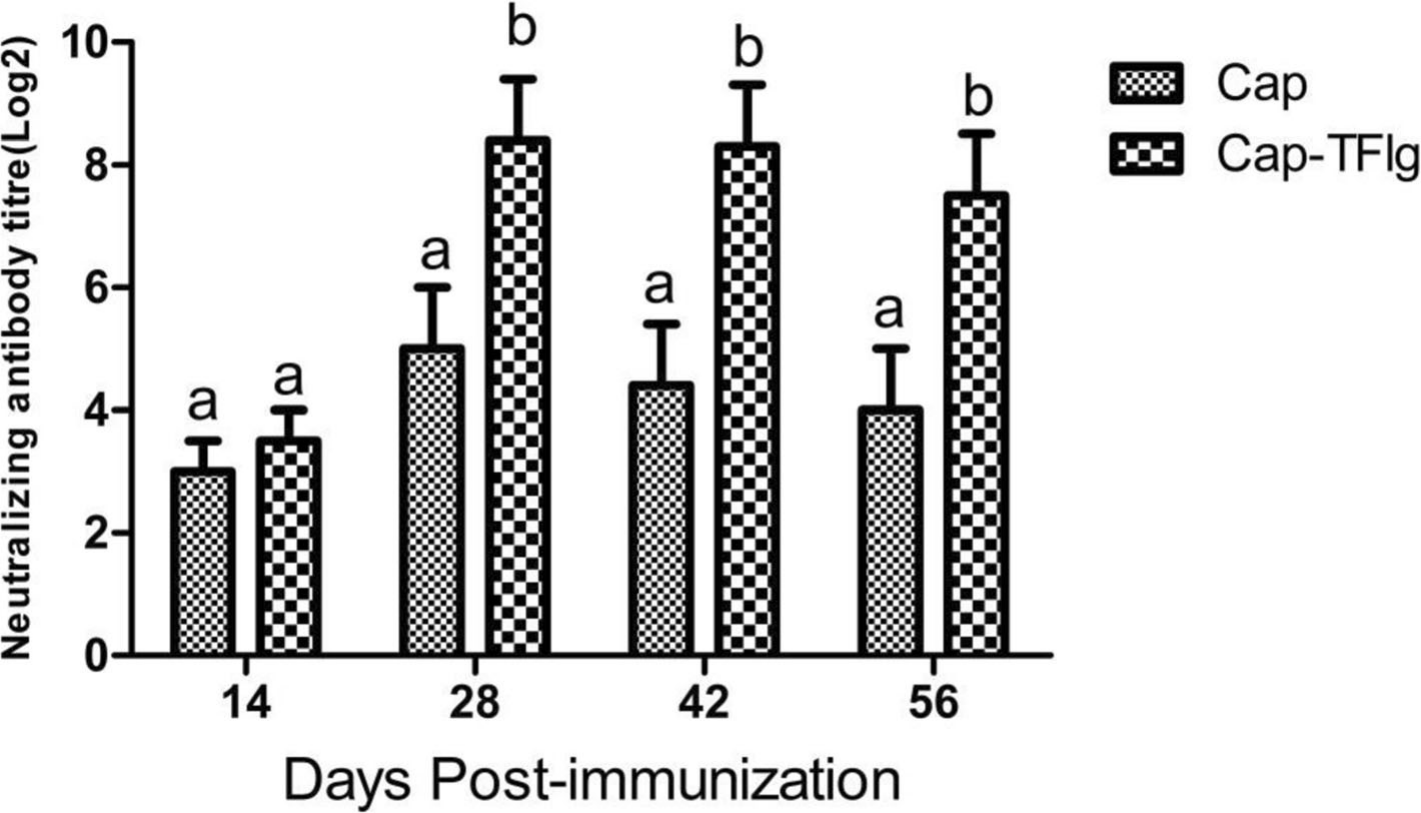
Detection of neutralizing antibodies in the sera Neutralizing antibodies were determined in the serum samples of mice (n = 5 per group) at 14, 28, 42 and 56dpi. The neutralizing antibody titers were calculated as the log2 of the reciprocal of the highest serum dilution that was capable of completely blocking PCV2-infection in PK-15 cells, and are expressed as mean ± SD of triplicate measurements. The data sets labeled with different superscript letters (a, b, and c) are statistically different from each other (P < 0.05)

As shown in Fig. 5, the neutralizing antibody titers in the Cap and Cap-TFlg vaccinated group were 2.98 and 3.46 at 14 dpi, respectively. The antibody titers increased to 5.18 and 8.55 at 28 dpi, respectively. At 28 dpi, the neutralizing antibody titers in the Cap-TFlg vaccinated groups were significantly higher than those in the Cap vaccinated group. The sera of mice in the PBS group did not develop neutralizing antibodies during the entire experiment.

### Cellular immune responses

**Fig. 6:**
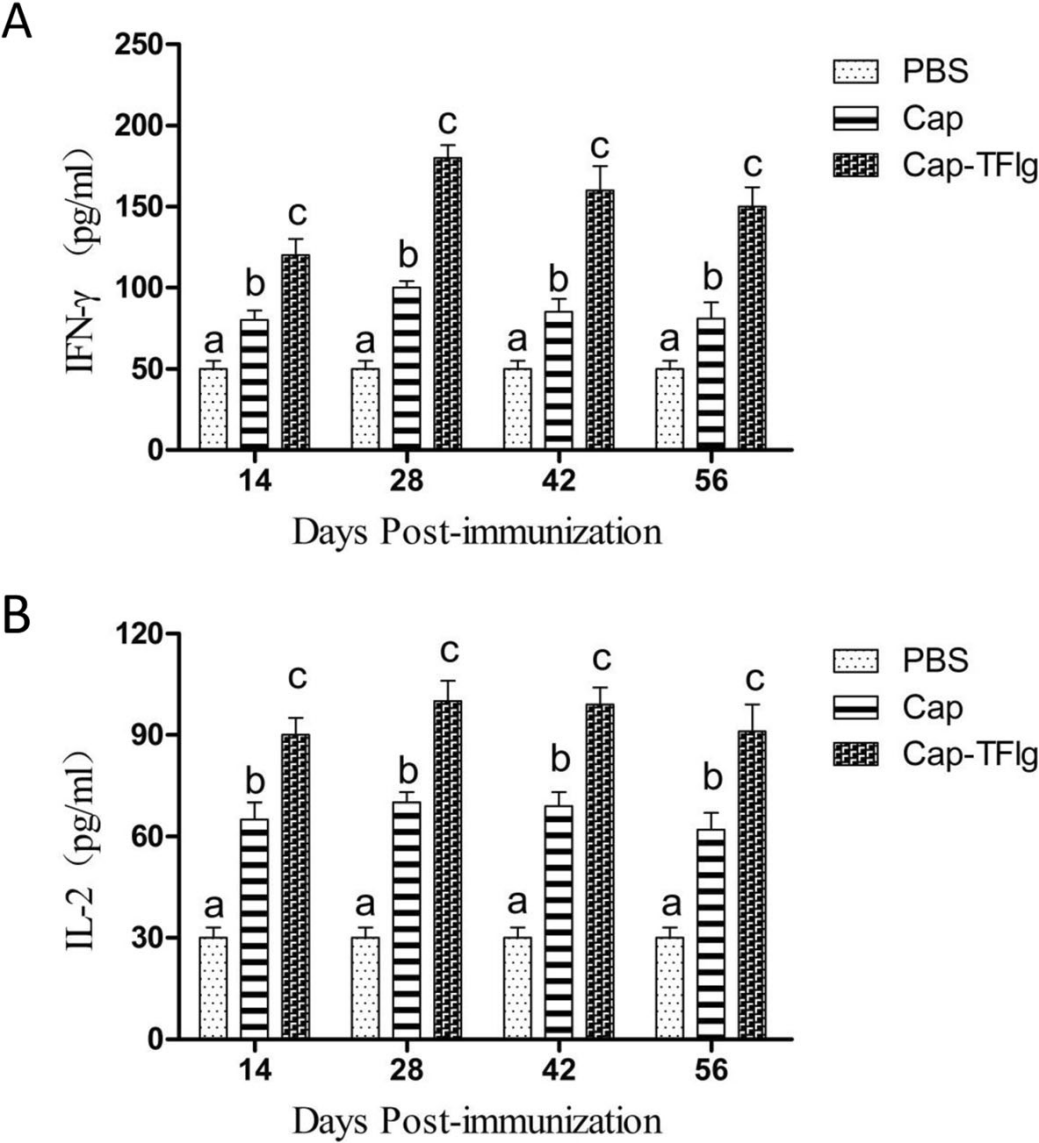
PCV2-specific cytokine production in the sera At 14, 28, 42 and 56dpi, the levels of IFN-γ (**a**) and IL-2 (**b**) in the serum samples were determined using a Bio-plex Pro mouse Cytokine 23-plex array kit. Data are expressed as mean ± SD of triplicate measurements. The data sets labeled with different superscript letters (a, b, and c) are statistically different from each other (*P* < 0.05)

A Bio-plex Pro Mouse Cytokine 23-Plex array kit was used to determine the production of IFN-γ and IL-2 in serum samples at 14 and 28 dpi. As shown in Fig. 6a and b, the concentrations of IFN-γ and IL-2 in the two immunized groups were significantly higher than those in the PBS group (*P* < 0.05). Furthermore, levels of IFN-γ and IL-2 expressed in the mice in the Cap-TFlg vaccinated group were significantly higher than those in the other groups (*P* < 0.05).

### Viremia

To examine the development of viremia, viral DNA extracted from sera was detected by routine PCR. Among the three mice inoculated with PBS, three developed viremia at 7 and 14 dpc, two developed viremia at 21 dpc, and viremia was completely undetectable at 28 dpc. In contrast, none of the mice in the Cap and Cap-TFlg vaccinated groups developed viremia throughout the challenge experiment.

### Quantification of PCV2 load in lungs

**Fig. 7:**
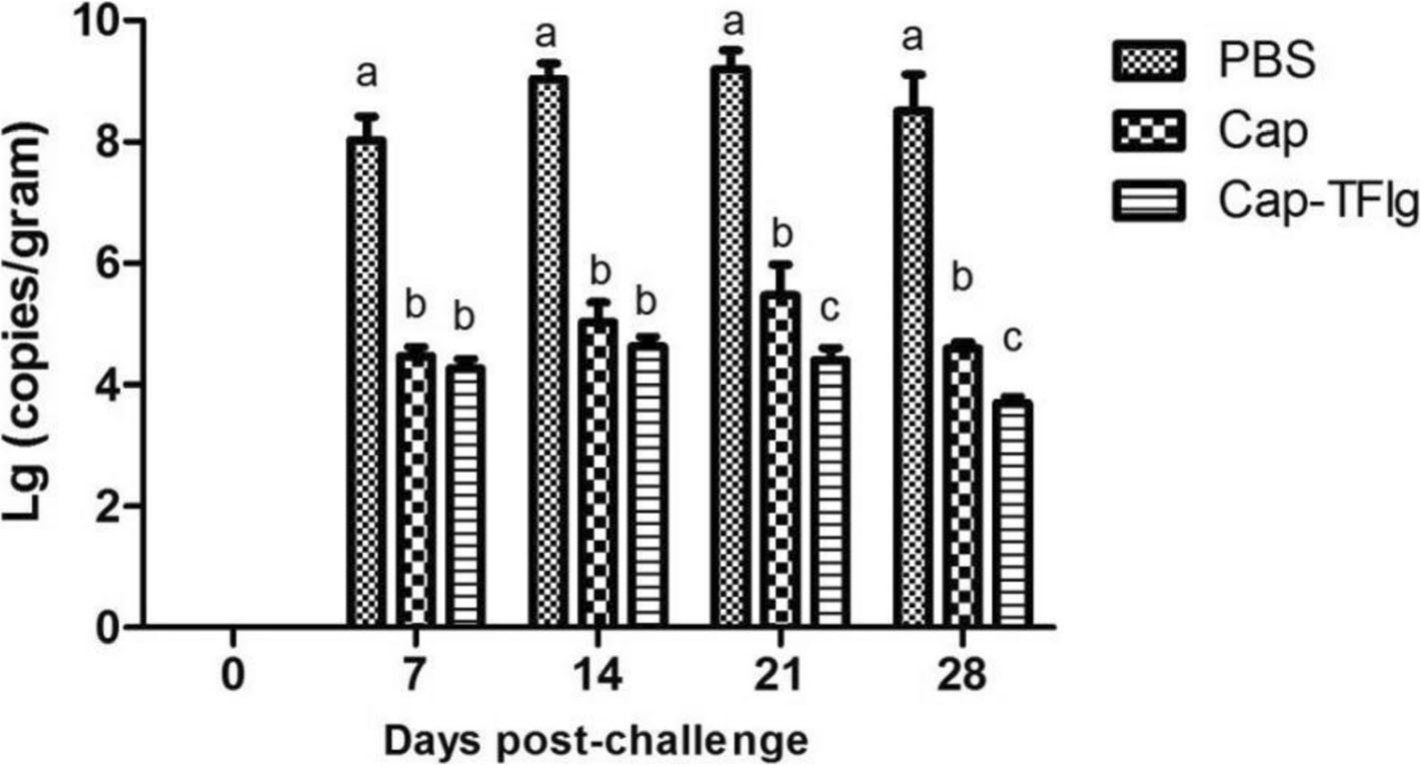
Quantification of PCV2 viral loads in lung samples of mice (dpc) Lung samples were collected from the mice at 0, 7, 14, 21, and 28 dpc, and their viral loads were quantified by real-time PCR. All lung samples collected at 0 dpc were negative. The data sets labeled with different superscript letters (a, b, and c) are statistically different from each other (*P* < 0.05)

To investigate whether vaccination reduced PCV2 loads in the lungs after the challenge, Five mice been euthanized Every time In 0 days, 7 days, 14 days, 21 days and 28 days, respectively, and tissue was collected and viral DNA extracted from lung samples post challenge was detected by real-time PCR. As shown in Fig. 7, the viral loads in lung samples of the two immunized groups were significantly lower than those in the PBS group during the challenge experiment (*P* < 0.05). At 7 and 14 dpc, the viral loads in the lung samples from the Cap-TFlg vaccinated group were lower than those in the Cap group, whereas there was no apparent difference between the two groups (*P* > 0.05). A significantly lower viral load was present in the Cap-TFlg vaccinated group as compared with that of the Cap vaccinated group at 21 and 28 dpc (*P* < 0.05).

## Discussion

Previous studies reported that Cap protein-based VLP vaccines induced protection against a PCV2 challenge [7, 8, 21, 22]. In addition, two reports showed that a flagellin-related peptide, 9Flg, enhanced antigen-specific and antitumor immunity [18, 23]. The previous work has confirmed that PCV2 virions surface displaying 27 aa peptide at C terminal of Cap could replicate as wild-type virions, which provided the clues that C terminal of Cap could display 27 aa peptide without influence the virions formation [24]. Based on this reference, the C terminal of Cap could be fused with short peptide without influence the Cap VLPs formation and stability. In addition, the Cap VLPs could induce 100% protection no matter what system for Cap expression. In this study, a recombinant VLP vaccine containing the TFlg gene at the C terminal of the Cap gene of PCV2 was constructed. The results of the present study suggest that its ability to protect against PCV2 is promising. The TFlg-containing Cap protein (Cap-TFlg) was identified by Western blotting and TEM. The results showed that the Cap-TFlg protein self-assembled into VLPs, with a size similar to that of the PCV2 Cap protein. A previous study showed that the C terminal of Cap was able to tolerate insertion of foreign epitopes of at least 27 amino acids [24, 25]. These findings are supported by those of the present study, which showed that TFlg insertion did not affect the formation of VLPs [26].

The mouse has been widely used as an infection model to elucidate the in vivo behaviors of virus-host interactions [27–29]. PCV2 has been shown to replicate and spread in BALB/c [24, 30],and Kunming mice [32, 33] .PCV2 nucleic acids can be detected in lymphoid tissues, the liver, epithelial cells, and the thymus [31]. Furthermore, the virus can be transmitted directly from mouse to mouse by contact, and it causes vertical infection through the placenta [32, 34–36]. Microscopic lesions in PCV2-infected mice are characterized by the expansion of germinal centers in lymphoid organs, with large numbers of histiocytic cells and lymphoblasts, apoptosis of histiocytic cells in germinal centers, and mild lymphoid depletion of the paracortex.

Although some research groups have reported that mouse models provide only limited utility in advancing the understanding of PCVD/ PCVAD [30, 37, 38] and that the ORF3 protein has very limited pathogenicity in its primary host [39],mouse models are useful for the study of immune responses to PCV2 in the context of an animal host.

In the present study, incorporation of TFlg into VLPs induced antibody and neutralizing antibody responses that were higher than those induced by standard VLPs. As shown in Fig. 4, the PCV2-specific antibody titers in the Cap-TFlg vaccinated group were significantly higher than those in the PBS group and Cap vaccinated group after 14 dpi (*P* < 0.05). Furthermore, the neutralizing antibody titers in the Cap-TFlg vaccinated group were significantly higher than those in the Cap vaccinated group at 28 dpi (Fig. 5). The results reveal that TFlg is a potent adjuvant for promoting humoral immunity.

Previous reports demonstrated that cell-mediated immunity specific to PCV2 might contribute to clearance of PCV2 [40, 41]. In the present study, the levels of IFN-γ and IL-2 in the serum of vaccinated mice were detected to evaluate cellular immune responses. The results indicated that the Cap-TFlg vaccine induced significantly higher levels of IFN-γ and IL-2 production than the Cap vaccine (*P* < 0.05). As IFN-γ and IL-2 are representative Th1-biased cytokines, the findings suggest that TFlg might enhance Th1-biased immune responses to the PCV2 Cap vaccine.

Previous studies investigated the viral distribution in Kunming mice infected with PCV2b and found that the lung contained PCV2 antigen-positive cells [32, 33]. Therefore, in the present study, the lung of challenged mice was selected as the target organ for detection of PCV2 genomic DNA and analysis of the protective effects of Cap-TFlg vaccines against PCV2 infection. As shown by the results, following the challenge, the two immunized groups did not develop viremia, as shown by sera samples. In addition, the Cap-TFlg protein significantly reduced the viral loads in lung samples as compared with the Cap protein. These results showed that Cap-TFlg provided better protection against the challenge and that TFlg enhanced the protective immune responses of the VLP-based PCV2 vaccine. As PCV2 VLPs were high positive charge (PI> 10) and high stability (over 3 months in room temperature) in high salt and near-neutral pH solution [42],we did not clearly describe the conditions of VLPs stability in this study. There was only one glycosylation site (143-145aa) on the PCV2 cap protein, and the amino acid sequence was NYS [43]. It was found that this glycosylation site mutation enhanced the specific immune response of DNA vaccine in mice. However, We compensated for the decreased immunogenicity by increasing the injection of PCV2 VLP, which was economical for the prokaryotic expression system.

For the efficiency assessment of PCVAD vaccine, the Kunming mice have been widely used as PCV2 infected animal models instead of pigs [33, 34]. The Salmonella typhimurium flagellin as TLR5 ligand has broad adjuvant activity without species specific. In this study,only females mouse were used,the subsequent experiments have added males panel and found no significant difference in antibody levels between males and females group. The Lung, spleen and lymph node are the most important organs for the infection of circovirus, In this experiment, To enable evaluation of vaccine candidates and investigate whether vaccination reduced PCV2 loads in the organs after the challenge, We just choose the lungs in the detection. If conditions permit, We will test the PCV2 loads in lymph nodes, liver, lung and spleen of pigs in subsequent experiments. In addition,as most of pigs in china are PCV2 positive,the PCV2 negative pigs are very difficult to find except for in PCVAD vaccine manufacturing enterprises. Due to limited conditions, we have proved in mice that the preliminary adjuvant can induce better cellular immunity. In the later stage of the experiment, we will screen negative pigs and supplement the experimental data in pigs.

## Conclusion

In conclusion, this is the first study to construct a prokaryotic vector expressing PCV2 Cap protein fused with adjuvant TFlg for the generation of recombinant VLPs. The results demonstrated that the recombinant Cap-TFlg protein not only generated VLPs but also enhanced humoral and cellular immune responses against a PCV2 challenge in mice. Moreover, chimeric VLPs containing TFlg were more efficient at eliminating the virus, indicating that TFlg may be a potent adjuvant for PCV VLP-based vaccines, which may provide a new strategy in the design of PCV2 vaccines.

## Methods

### Construction of expression plasmids for SUMO-cap and SUMO-cap-TFlg fusions

The gene sequence coding the PCV2 cap protein (Genbank JF272498.1) was codon optimized for expression in *E. coli* (details can be found in the Additional file 1). The gene was come from PCV2 strain SH (2b). and PCV2 SH (2b) strain was used for the virus neutralization assay.

The primers used in this study are listed in Table 1.

**Table 1:** Primers used in this study

Genes	Name	Primer Sequence (5′-3′)
SUMO	SUMO-F	GCCATGGGTCATCACCATCATCATCACGTGGGTCGGACTCAGAAG
	SUMO-R	ACCTCCAATCTGTTCGCGGTGAGCCTCAAT
	Cap-F	GAACAGATTGGAGGTATGACCTACCCGCGTCGTCG
Cap	Cap-R1^a^	CTCGAGTTACGGGTTCAGCGGAGGGTC
	Cap-R2^b^	CGGGTTCAGCGGAGGGTCCTTCA
TFlg	TFlg-F	ACCCTCCGCTGAACCCGATCAACAACAACCTGCA
	TFlg-R	GCCGCTCGAGTTAGGAGTCGAGGTCAGACTGG

^a^ Primer used to clone the SUMO-Cap gene; ^b^ Primer used to clone the SUMO-Cap-TFlg gene; The underlined sequences represent the restriction enzyme site (NcoI and XhoI). The sequences in lowercase letters indicate the His6 tag. The sequences in bold indicate the stop codon

The SUMO gene was amplified from a pET SUMO vector with primers SUMO-F and SUMO-R. An 81-bp truncated form of the flagellin gene (TFlg) encoding amino acids 85–111 of the mature flagellin FliC from *S. typhimurium* (GeneBank D13689) was amplified from a pMD18T-FliC vector with primers TFlg-F and TFlg-R. As shown in Fig. 1, the SUMO-Cap and SUMO-Cap-TFlg DNA fragments were generated by overlap extension PCR as described previously [44] and then cloned into a pET32a vector. The resultant plasmids were verified by DNA sequencing.

### Expression and purification of SUMO-cap and SUMO-cap-TFlg fusion proteins

The positive plasmids were transformed into *E. coli* BL21 (DE3) cells for protein expression. The transformants were grown at 37 °C in LB liquid medium containing 100 μg/mL of ampicillin. When the optical density (OD_600_) reached 0.6, 0.4 mM IPTG was added to induce protein expression. After an additional 6 h of cultivation at 30 °C, the cells were harvested by centrifugation at 6000 g for 10 min at 4 °C [45]. The cell pellets were resuspended in 50 mM Tris-HCl buffer (1.0% Triton X-100, pH 8.0) and then disrupted using sonication, followed by centrifugation to remove cell debris. The supernatants were loaded onto a Ni-NTA column equilibrated with 20 mM Tris-HCl buffer (150 mM NaCl, 20 mM imidazole, pH 8.0). Bounding protein was eluted with 500 mM imidazole in the same buffer. Fractions containing the fusion proteins were collected and concentrated at 4 °C.

### Cleavage of fusion proteins

The SUMO-Cap and SUMO-Cap-TFlg fusion proteins were incubated with SUMO protease for 16 h at 4 °C, and the mixture was then passed through an Ni-NTA column to remove uncleaved SUMO fusion protein, SUMO tag, and SUMO protease. The untagged Cap and Cap-TFlg proteins were collected in the flow-through fraction and concentrated using an ultrafiltration tube. Finally, the protein was dialyzed against PBS (pH 7.4) and further analyzed by Western blotting. The concentration of Cap and Cap-TFlg proteins was quantified using a BCA protein assay.

### Removal of endotoxin

To remove the endotoxin, 1.5% Triton-X114 was added to the redissolved solution, followed by cold shock for 6–8 h at 4 °C or immersion in an ice bath for 1 h. After processing, the sample temperature slowly recovered to around 30 °C, and it was then centrifuged at 10,000 rpm for 30 min at 30 °C. The centrifuge tube was gently removed after centrifugation, and slowly sucking the clear liquid. Then, 1.2% Triton-X114 was added to the sample, and the above steps were repeated a third time: Addition of 1% Triton-X114 to the second step sample, repeat the above steps and determine endotoxin quantitively.

### Western blotting

The purified Cap and Cap-TFlg proteins were separated by 15% SDS-PAGE and then transferred onto polyvinylidene fluoride membranes. The transferred membrane was blocked and then incubated with the recommended dilution of rabbit anti-Cap antibody (1:200, made in our lab), followed by incubation with 1:8000 diluted HRP-conjugated goat anti-rabbit IgG (Sungene, Tianjin, China). The protein bands were visualized using an enhanced HRP-DAB Chromogenic Substrate Kit (Tiangen, Beijing, China).

### Detection of VLP formation

The purified Cap and Cap-TFlg proteins were placed on a carbon-coated copper grid, dried with filter paper, negatively stained with 2% phosphotungstic acid, and examined by a transmission electron microscope (HT7700, HITACHI) at an acceleration voltage of 120 kV.

### Immunization of animals

The purified Cap and Cap-TFlg proteins were adjusted at a final concentration of 0.5 mg/mL. All animal protocols were performed in accordance with the guidelines of the ethical committee of Northwest Agriculture and Forestry University. The mice were purchased from Wuhan Biological products Research Institute Co., Ltd. Forty-five female Kunming mice (6-week-old, SPF mice, BALB/c) were randomly assigned into 3 groups, each comprising 15 mice. The mice in group A were intramuscularly immunized with 100 μg of Cap protein in a final volume of 200 μL; mice in group B were inoculated intramuscularly with 100 μg of Cap-TFlg protein following the protocol described above; and mice in group C received an intramuscular injection with 200 μL/mouse of PBS as a negative control. The mice were boosted at 2-week intervals using the same inoculation protocols. Two weeks after the second immunization, all the mice were challenged intraperitoneally with 10^4.5^ TCID_50_ PCV2 SH strain. Serum samples were collected on days 0, 7, 14, 21, 28, 35, 42 and 56 after the first immunization for detection of antibodies to PCV2. Following the viral challenge, three mice from each group were euthanized weekly, and serum and lung samples were obtained for viral nucleic acid and viral load analyses.

### Detection of humoral immune responses

Serum samples from five mice in each group were collected weekly after the first immunization. The levels of PCV2-specific antibody and PCV2-neutralizing antibody were analyzed to determine the humoral immune response.

An indirect ELISA was used to detect PCV2-specific antibody [21]. In brief, 0.1 μg of the recombinant Cap protein diluted in a coating solution (bicarbonate buffer, pH 9.6) was coated on 96-well plates. Serum from mice sera in each group (1/100) was used as the primary antibody, and HRP-conjugated goat anti-mouse IgG (1/5000) (Sungene, Tianjin, China) was used as the secondary antibody. HRP activity was then detected using TMB substrate, and the OD values were measured at a wavelength of 450 nm.

Virus neutralizing antibody was detected as described previously [30]. Briefly, fifty microlitres of serum tested were serially two-fold diluted in 96-well plates, from 1:2 to 1:4096 in complete DMEM (Dulbecco’s Modified Eagle Medium), supplemented with 5% foetal bovine serum (FBS), 1% L-glutamine, 10,000 U/ml of penicillin, 50 μg/ml streptomycin and 3% non-essential amino acids). From a PCV2 stock (SH strain), produced as previously described and adjusted in complete DMEM to abovementioned concentrations, 50 μl were added to each well. After one hour of mixture incubation, 2*10^4^ freshly tripsinised Swine Kidney (SK) cells were added to each well and incubated for 36, 42 or 72 h at 37 °Cin 5% CO_2_ atmosphere. Then, cells were washed twice with PBS and fixed with cold absolute ethanol at − 20 °C for 30 min. Plates were then incubated for 1 h at 37 °C with a hyperimmune serum against PCV2 diluted 1:200 in phosphate-buffered saline containing 0.1% Tween 20 (PBS-Tween) and 1% bovine serum albumin. After washing with PBS-Tween, peroxidase-labelled protein A (0.6 μg ml ^− 1^) was added and plates were incubated for 1 h at 37 °C. Finally, plates were washed with PBS-Tween and amino-ethyl-carbazole was added to reveal the reaction. All samples were tested in duplicate. In each plate, a serum sample with a known neutralizing antibody titre was included as a positive control, and complete DMEM was used as negative control (one row of wells). Finally, the viral inoculum used in each VNT was titrated to ascertain the accuracy of the viral concentration.

### Detection of cellular immune responses

To investigate the cellular immune response induced by the recombinant proteins, serum samples were collected at 14 and 28 days postimmunization (dpi). A Bio-plex Pro Mouse Cytokine 23-Plex array kit (Bio-Rad) was the used to measure levels of IFN-γ and IL-2 in the samples, according to the manufacturer’s protocols. All samples were analyzed in triplicate, and the data were presented as concentrations (pg/mL).

### Detection of PCV2 viremia in serum by PCR

To investigate the development of viremia in serum, total DNA was extracted from serum samples collected weekly post challenge using a DNA extraction kit (Takara, Dalian, China). The DNA was then used as a template for PCR amplification with the following primers: 5′-GTTTACATAGGGGTCATAG-3′ (Cap-up) and 5′-TGTGCCCTTTGAATAC (Cap-down). Routine PCR was performed as described previously [25].

### Detection of PCV2 nucleic acids in lungs by real-time PCR

Lung samples were collected 0, 7, 14, 21, and 28 dpc for quantification of PCV2 nucleic acids.the mice were euthanized by the physical method of cervical spine fracture, The experimenter fixed the back of the mouse’s head with tweezers and clipped the tail with the right hand then pull back and up, the mouse’s cervical vertebra dislocated and died instantly. Viral DNA extracted from lung samples using a DNeasy Blood & Tissue Kit was used as a template for quantitative real-time PCR. Cap-up and Cap-down primers were used to amplify part of ORF2 of PCV2 (135 bp). Real-time PCR was performed using a Bio-Rad iQ5 (Bio-Rad, USA), and 12.5 μL of FastStart Essential DNA Green Master (Roche) was used in each reaction. Serial dilutions of plasmid pMD18T-Cap were used to generate a linear standard curve. The mean of the logarithmic PCV2 copy number per gram of lung (Lg copies/g) was calculated for comparisons among the different groups.

### Storage of PCV2 VLPs

The addition of 15% trehalose in storage buffer promoted long-term preservation of PCV2 VLPs, thereby facilitating maintenance of particle morphology, without impairing stability during vaccine production, storage, and transportation.

### Statistical analysis

The experimental data were analyzed using a one-way analysis of variance (ANOVA), combined with Tukey’s post hoc test. *P* < 0.05 was considered statistically significant.

## Supplementary information

**Additional file 1.** The gene sequence of the PCV2 cap protein.

## Data Availability

The datasets used and/or analysed during the current study are available from the corresponding author on reasonable request.

## Abbreviations

9Flg: 9flagellin-related peptides
Cap: Capsid protein
dpc: days post viral challenge
dpi: days post immunization
IFN-γ: Interferon-γ
IL-2: Interleukin-2
IPTG: Isopropyl β-D-Thiogalactoside
LB: Lysogeny broth
mM: mmol/L
ORFs: Open reading frames
PCV2: Porcine circovirus type 2
PCVADs: PCV2-associated diseases
rpm: revolutions per minute
SUMO: Small ubiquitin-like modifier
TEM: Transmission electron microscopy
TFlg: Truncated form of flagellin
VLPs: Virus-like particles
