# Supplementary material for: Incorporation of a truncated form of flagellin (TFlg) into porcine circovirus type 2 virus-like particles enhances immune responses in mice
Source: BMC Vet Res. 2020 Feb 7;16:45. doi: 10.1186/s12917-020-2253-6 (PMC7006081; doi:10.1186/s12917-020-2253-6)
Supplement: Supplementary file 1 — Additional file 1. The gene sequence of the PCV2 cap protein. [file 12917_2020_2253_MOESM1_ESM.docx]

Supplementary file ATGACGTATCCAAGGAGGCGTTTCCGCAGACGAAGACACCGCCCCCGCAGCCATCTTGGCCAGATCCTCCGCCGCCGCCCCTGGCTCGTCCACCCCCGCCACCGTTACCGCTGGAGAAGGAAAAATGGCATCTTCAACACCCGCCTCTCCCGCACCTTCGGTTATACTGTCAAGAAAACCACAGTCAGAACGCCCTCCTGGGCGGTGGACATGATGAGATTTAATATTAATGATTTTCTTCCCCCAGGAGGGGGCTCAAACCCCCGCACTGTGCCCTTTGAATACTACAGAATAAGGAAGGTTAAGGTTGAATTCTGGCCCTGCTCCCCAATCACCCAGGGTGACAGGGGAGTGGGCTCCACTGCTGTTATTCTAGATGATAACTTTGTAACAAAGGCCAATGCCCTAACCTATGACCCCTATGTAAACTACTCCTCCCGCCATACCATAACCCAGCCCTTCTCCTACCACTCCCGGTACTTTACCCCGAAACCTGTCCTTGATAGGACAATCGATTACTTCCAACCCAATAACAAAAGAAATCAACTCTGGCTGAGACTACAAACTACTGGAAATGTAGACCATGTAGGCCTCGGCACTGCGTTCGAAAACAGTATATACGACCAGGACTACAATATCCGTATAACCATGTATGTACAATTCAGAGAATTTAATCTTAAAGACCCCCCACTTAACCCTTAA
